# Gene–environment interactions and their impact on human health

**DOI:** 10.1038/s41435-022-00192-6

**Published:** 2022-12-30

**Authors:** Samuel J. Virolainen, Andrew VonHandorf, Kenyatta C. M. F. Viel, Matthew T. Weirauch, Leah C. Kottyan

**Affiliations:** 1 Division of Human Genetics, Center for Autoimmune Genomics and Etiology, Cincinnati Children’s Hospital Medical Center, 3333 Burnet Ave., Cincinnati, OH 45229 USA; 2 Immunology Graduate Program, University of Cincinnati College of Medicine, 3230 Eden Ave, Cincinnati, OH 45229 USA; 3 Divisions of Biomedical Informatics and Developmental Biology, Cincinnati Children’s Hospital Medical Center, 3333 Burnet Ave., Cincinnati, OH 45229 USA; 4 Department of Pediatrics, University of Cincinnati College of Medicine, 3230 Eden Ave, Cincinnati, OH 45229 USA; 5 Division of Allergy and Immunology, Cincinnati Children’s Hospital Medical Center, 3333 Burnet Ave., MLC 15012, Cincinnati, OH 45229 USA

**Keywords:** Population genetics, Immunology

## Abstract

The molecular processes underlying human health and disease are highly complex. Often, genetic and environmental factors contribute to a given disease or phenotype in a non-additive manner, yielding a gene–environment (G × E) interaction. In this work, we broadly review current knowledge on the impact of gene–environment interactions on human health. We first explain the independent impact of genetic variation and the environment. We next detail well-established G × E interactions that impact human health involving environmental toxicants, pollution, viruses, and sex chromosome composition. We conclude with possibilities and challenges for studying G × E interactions.

## Introduction

For centuries, clinicians and scientists have sought to understand the etiology of disease. While some diseases can be traced back to a single factor, the etiology of complex diseases is more difficult to discern, in part due to the combinatorial nature of various contributing factors. One such factor contributing to disease risk is an individual’s genetics, with some individuals inheriting specific genetic variants that either (1) directly trigger disease pathogenesis or (2) work in concert with other factors and/or other genetic variants to increase disease risk. In many cases, Environmental exposures, defined here as pathogens, chemicals, and additional external factors, have also been shown to contribute to disease. While epidemiological studies can identify associative relationships between exposure to environmental factors and disease pathogenesis, not all individuals who are exposed to a specific environmental factor develop disease. Likewise, not all individuals who inherit particular genetic variants develop disease. For the vast majority of diseases, it is apparent that combinations of synergistic or antagonistic factors are important to disease risk. Such “Gene by Environment” (G × E) interactions are the focus of this review.

**Fig. 1:**
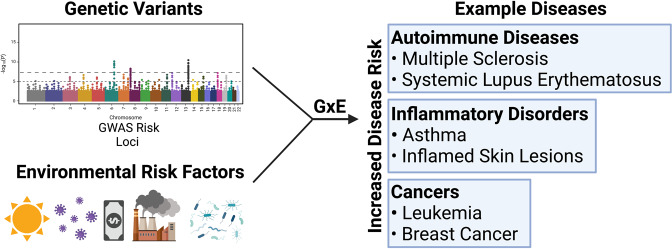
Gene × environment (G × E) interactions involve synergy between environmental risk factors and genetic variants. Some G × E interactions can increase the risk of disease. A model of G × E interaction originally defined in Ottman [8] and further refined in Kauffmann and Demenais [9] is depicted where the genetic risk variants and one or more environmental risk factor synergistically affect disease risk.

Prior reviews have described G × E interactions in nonhuman organisms such as yeasts [1], or in specific human disease contexts, such as inflammatory diseases or particular psychological conditions [2–7]. Others have focused on models of GxE interactions, such as Ottman [8] and Kauffman and Demenais [9] who collectively proposed four models for G × E interactions: (1) the risk genotype exacerbates the effect of the environmental risk factor; (2) exposure to the environmental risk factor exacerbates the effect of the risk genotype; (3) both the environmental risk factor and risk genotype are required to increase disease risk, and (4) the environmental risk factor and the genotype each have some effect on disease risk, and risk is higher when they occur together than when they occur alone. This review focuses on the latter model, where a synergistic relationship between environmental risk factors and genetic factors increases disease risk (Fig. 1). Herein, we broadly review G × E in the context of human health with a focus on how particular genetic and environmental factors synergistically increase disease risk. We first describe instances where genetic and environmental risk factors can independently potentiate disease. We then examine how these two factors can work together to increase disease risk.

### Genetic etiology of disease

In many cases, genetic variants impact phenotypes that contribute to disease pathology. Historically, family studies have been used to measure the contribution of genetics to a particular trait through studies comparing disease risk in monozygotic twins (100% shared genetic identity), dizygotic twins (50% shared genetic identity), and siblings (also 50% shared genetic identity) [10]. Significant differences between monozygotic and dizygotic disease concordance establish strong genetic heritability. For example, three independent twin studies have identified a monozygotic twin concordance for Crohn’s disease of ~50% compared to ~3–4% disease concordance in dizygotic twins [11–13]. The difference in concordance of monozygotic and dizygotic twins also highlights the possible role of epistatic risk factors that require interactions between multiple genetic variants to increase disease risk [14]. The over ten-fold increase in Crohn’s disease concordance in siblings with identical DNA sequence compared to siblings sharing 50% genetic identity indicates a large genetic component to disease risk.

Diseases with a genetic component can be monogenic (i.e., caused by a single rare mutation), complex (i.e., caused by the cumulative effect of multiple genetic events and/or the environment), or both. For example, cystic fibrosis is a monogenic condition caused by a mutation in the *CFTR* gene [15, 16], with additional genetic variants impacting disease presentation and severity [17]. However, many “causal” disease mutations are incompletely penetrant, with some individuals carrying the mutation expressing various degrees of symptoms and some individuals not expressing the disease at all [18]. For example, only 10–30% of people with damaging mutations in the gene encoding complement component 2 (C2) develop systemic lupus erythematosus (lupus) [19]. Instead, most disease manifestations are the result of complex etiologies, with many relatively common genetic polymorphisms (i.e., allele frequencies greater than 1%) contributing to disease risk in an additive manner [20]. Indeed, most patients with lupus do not have monogenetic disease [21], with the most common form of monogenic lupus (mutations in the *TREX1* gene) contributing to only 0.5 to 2% of adult lupus cases [22].

#### Identifying genetic risk loci

Genome-wide association studies (GWAS) have emerged as the predominant tool for the systematic, genome-wide identification of disease-associated genetic risk variants. Such studies genotype thousands of cases and controls to identify statistically significant genetic associations between particular variants and a given disease phenotype [23]. The most recently published GWAS “catalog” contains over 5000 independent GWAS datasets that describe more than 70,000 variant-trait associations [24]. While such studies are helpful for identifying disease risk loci for further functional analysis, they have shortcomings. First, study participant environments are not standardized, and thus, potential environmental effects are not well controlled. Second, only a small fraction of the variants identified in a given GWAS are causal, due to linkage disequilibrium; specifically, the particular variant(s) directly studied (“tagged”) in the GWAS may simply be in strong linkage disequilibrium with the variant that is functionally influencing the phenotype [25]. Finally, GWAS to date have focused disproportionately (>78%) on individuals of European descent [26]. Additional studies focused on nonwhite populations are thus needed to obtain a clearer picture of the disease spectrum across ancestries [27, 28]. Despite these limitations, the increasing availability of GWAS data has enabled researchers to pursue novel hypotheses and design targeted studies to further investigate functional roles between a particular variant and its associated phenotype.

#### Functional interpretation of genetic risk loci

While GWAS have identified many genetic risk loci, additional studies are required to elucidate the causative molecular mechanisms underlying disease. Genetic variants identified in GWAS can increase disease risk through multiple means, including changing the amino acid of a protein, altering gene regulatory mechanisms, impacting RNA splicing, and affecting translation rates. For example, the identification of loss-of-function nonsynonymous coding genetic variants within the filaggrin (*FLG*) gene in ~10% of atopic dermatitis patients [29] led to further studies revealing the role of FLG in the development of a healthy epidermis [30, 31] and atopic dermatitis pathogenesis [32]. In particular, studies in cells from patients with *FLG* mutations and in mice lacking a functional FLG helped shape the current hypothesis that defects in the skin barrier lead to allergic sensitization and the progression of atopic dermatis in infancy toward asthma and allergic disease in later childhood, commonly known as the atopic march [33]. More recent studies have focused on the role of *FLG* mutation-mediated skin barrier defects in skin dysbiosis and *Staphylococcus* colonization [34–36], illustrating how genetic studies can lead to previously unexplored avenues and, ultimately, targeted studies to ascribe functional contributions of variants to disease processes.

Mutations at the *FLG* locus demonstrate how a change in the genetic code can contribute to disease processes by altering the amino acid sequence of a protein, which may affect its structure and/or function as a consequence. However, it should be noted that variants do not need to occur in coding regions to elicit a phenotype. In addition to coding mutations, variants in noncoding regions also make substantial contributions to disease risk by altering gene regulatory mechanisms. Indeed, GWAS variants for many diseases are highly enriched within noncoding genomic regions [37]. Such variants likely alter gene regulatory mechanisms, leading to genotype-dependent variability in gene expression levels that contribute to disease risk [37, 38], often by altering transcriptional elements (e.g., promoters or distal regulatory elements such as enhancers) or posttranscriptional elements (e.g., regions controlling mRNA splicing or mRNA localization) [39]. One important way in which noncoding variants affect regulatory element function is through the alteration of transcription factor (TF) DNA binding interactions. Both amino acid-altering variants located within the TF proteins themselves and noncoding variants located within TF genomic binding sites can alter TF-DNA binding specificity or affinity [40–42]. For example, an obesity-associated intronic genetic variant in the *FTO* locus leads to genotype-dependent binding of the ARID5B TF, resulting in a doubling of the expression of the *IRX3* and *IRX5* genes, a genotype-dependent increase in energy-storing adipocytes, and a decrease in energy-dissipating adipocytes [43]. In addition to genetic variants that alter TF-based transcriptional regulatory mechanisms, genetic variants can also alter posttranscriptional regulatory mechanisms by affecting the binding of RNA binding proteins [44], microRNAs [45–47], or long noncoding RNAs [48].

Modern computational and statistical methods have enabled the robust assessment of genotype-dependent biology. Recently, quantitative trait locus (QTL) and allelic imbalance analyses have emerged as powerful tools for identifying genetic variants with genotype-dependent biological effects [49]. QTL studies identify genotype-dependent biology by quantitatively comparing a particular phenotype (e.g., the expression level of a gene) across many individuals as a function of the genotype of each individual [50]. Such associations can be used to describe the effects of genetic variants on cell biology, meaning that QTLs and GWAS data can collectively inform numerous aspects of disease mechanisms, including the nomination of likely causal disease variants [49]. For example, histone QTLs are highly enriched within autoimmune disease risk haplotypes in cell types relevant for disease etiology, implicating differential epigenetic mechanisms at multiple genomic loci [51].

GWAS identify groups of genetic variants that are inherited together as a genetic haplotype. Genetic association is often insufficient to narrow these variants down to the small group that actually change biological processes in a genotype-dependent manner. Additional computational methods can nominate causal disease variants by identifying risk alleles that alter the binding of particular regulatory proteins. An example of a current method is Measurement of Allelic Ratios Informatics Operator (MARIO), which identifies allele-dependent binding of regulatory proteins at heterozygous variants [52] by examining allelic imbalance in the reads of a functional genomics experiment. A substantial benefit of computational methods such as MARIO is that they can largely bypass the need for large sample sizes used in QTL analyses. However, such methods are also limited by their dependence on contextually-relevant datasets obtained through methods such as chromatin immunoprecipitation sequencing (ChIP-seq). While this information may be available for diseases that have been well-studied, sequencing datasets for regulatory proteins in less-common diseases may not be as abundant or available. Therefore, continued development of new analytical methodologies should be pursued in order to deliver the full benefits of large-scale data analysis to a broader range of topics in human health and disease. Collectively, QTLs, allelic imbalance analyses, and other advances in functional genomics methodologies have enabled researchers to go beyond risk variant identification to discover potential molecular mechanisms of disease underlying genetic associations.

### Environmental etiology of diseases

As a part of the environment, organisms are continuously exposed to a myriad of external factors that shape health and disease. Many environmental factors that individuals are routinely exposed to have been associated with disease risk, including the use and consumption of various substances, such as tobacco and alcohol, as well as exposure to ultraviolet light [53–56]. Environmental data can be collected prospectively through cohort studies or retrospectively through medical records, surveys, or government records. For example, the addresses reported by a child across numerous trips to the emergency department for asthma treatment can be converted into geocodes that allow quantification of exposure to air pollution near interstate highways and provide information on the median home price and salary [57–59].

The environment itself is constantly changing. Industrialization and urbanization adversely affect human health [60, 61], while climate change in turn threatens to alter the way humans live and interact with the environment [62]. The coronavirus disease-19 (COVID-19) pandemic has demonstrated how quickly the environment can change, with mitigation measures in place to combat the pandemic drastically changing the prevalence of influenza and other infectious diseases [63, 64]. In this section, we focus on major components of the environment and how they impact human health: the microbiome, pollutants and environmental toxicants, viral infection, climate change, and psychosocial and economic factors. While recent reports have comprehensively reviewed hallmarks of environmental insults (e.g., [65]), this section will highlight diseases that impact the immune system.

#### The microbiome

The microbiome includes all of the microbes that reside on and inside the human body. Contributing up to 100 trillion cells in adults, the microbiome plays critical roles in development, nutrition, digestion, and immunity [66–69]. In this section, we introduce the microbiome in the context of responding to external exposures, with an emphasis on disease risk.

The microbiome plays a critical role in immunity by providing protection against allergic disease. Lifestyle changes in recent decades, especially in high-income and industrialized countries, have led to a decrease in the incidence of infections while increasing the incidence of autoimmune and allergic diseases [70]. According to the hygiene hypothesis, this increase in allergic and autoimmune disease prevalence in recent decades is attributable to a decrease in infectious disease incidence, particularly in developed nations. While this concept is strongly supported by epidemiological data, the mechanisms driving this relationship are not as well characterized [71, 72]. Some gut microbiota confer protection against food allergen sensitization through the activation of genes involved in innate immunity [73, 74]. For example, germ-free mice transplanted with microbiota from a healthy infant were protected from an allergic response when challenged with cow’s milk allergen β-lactoglobulin, while the transfer of microbiota from infants allergic to cow’s milk resulted in a response [74]. Transcriptional analyses of intestinal epithelial cells from germ-free mice, healthy mice, and mice colonized with cow’s milk allergen identified unique transcriptomic signatures among the three groups. Notably, genes involved in epithelial repair and metabolism were expressed as a function of the type of colonization. In a separate study, Stefka et al. found an increased proportion of regulatory T cells in the colonic lamina propria and elevated concentrations of fecal immunoglobulin A of mice colonized with Clostridia-containing microbiota, mechanistically demonstrating how colonization with these microbiota provides protection against allergies [73]. Furthermore, intestinal epithelial cells from colonized mice also showed an increase in the expression of genes with functions in the innate immune system compared to germ-free mice. The identities of these genes further elucidate how the microbiota confer protection for disease. For example, regenerating islet-derived 3 beta (*REG3B*), which encodes an antimicrobial peptide that regulates mucosal microbiota composition, is upregulated in colonized mice. Moreover, clostridia colonization induces the immunological cytokine IL-22, resulting in intestinal epithelial cell-mediated production of antimicrobial peptides and protection of the intestinal epithelial barrier by increasing the number of mucus-producing goblet cells. These findings collectively suggest that microbiota may induce specific, immunologically relevant gene expression signatures that help protect against allergic disease.

While the underlying molecular and microbial mechanisms remain to be fully characterized, these exemplary studies highlight the critical role of commensal microbiota in shaping the immune system and, subsequently, their contributions to the modulation of susceptibility for multiple immune-related diseases. Notably, allergic diseases are just one of many phenotypes impacted by the microbiome, and the microbiome itself is affected by many other environmental factors including diet, birth mode, exposure to antibiotics, and age [75, 76].

#### Pollutants and environmental toxicants

Environmental exposures are complex and rely on several factors that include, but are not limited to, time, geographic region, and route of exposure. In the United States, residential proximity to sites containing environmental hazards has been associated with potential reduction in life expectancy [77] and multiple adverse health outcomes that have been extensively reviewed [78]. More broadly, the Lancet Commission on pollution and health reported that total pollution exposure was a leading risk factor for global estimated deaths in their analysis of the 2019 Global Burden of Diseases, Injuries, and Risk Factors Study data [79], attributing pollution to ~9 million deaths [79]. Given the magnitude of these disease burden estimates, understanding how environmental toxicants elicit adverse effects serves as a critical step for identifying populations at risk, reducing offending sources of emissions, and improving public health. In this section, we briefly highlight connections between environmental exposures and adverse health outcomes with an emphasis on ambient air pollution and water contamination.

The World Health Organization (WHO) has estimated that exposure to ozone and ambient air pollution, defined in their global burden estimates as particulate matter with a diameter less than or equal to 2.5 µm (PM2.5), can be attributed to 4 to 9 million global deaths annually [80]. The composition of PM2.5 is diverse and may depend on its source of introduction, with combustion-related activities of energy production, energy use, and industrial processes being notable sources of anthropogenic contribution [81]. Studies investigating the associations between PM2.5 and adverse health outcomes have implicated the particulate matter (and its composition [82]) in the initiation and progression of multiple diseases including cardiovascular disease [83–86], asthma [87–89], and lung cancer [90, 91], with the International Agency for Research on Cancer classifying both outdoor pollution and its relevant constituents as carcinogenic to humans [92]. This is particularly concerning given that the trend toward increased urbanization and anthropogenic activity has been positively correlated with changes in PM2.5 concentrations [93], reinforcing the need for air quality monitoring and pollution reduction initiatives.

Ingestion of contaminated food and water are additional routes of exposure, with UN-Water estimating that two billion people lacked safely managed drinking water services in 2020 [94]. Although many different types of contamination contribute to overall risk, here we introduce a subset of metals that have been consistently identified as significant environmental contaminants. In particular, arsenic is currently considered to be a global issue that may expose between 94 million and 220 million people to high concentrations of the metal through groundwater sources [95]. Chronic arsenic poisoning, also known as arsenicosis, frequently manifests in the form of skin lesions such as melanosis and keratosis [96], and has been causally associated with multiple cancers [97]. While arsenic contamination is often the result of natural processes, industrial wastewater and improper disposal methods of other metals have been attributed to the development of adverse health outcomes, including Minimata disease (methylmercury) [98] and Itai-itai disease (cadmium) [99].

Together, these examples provide a brief introduction to the intrinsic link that exists between environmental contaminants and human health. However, while some of the cases presented here implicate a single compound in elevated concentrations as the source of toxicity, this is not necessarily representative of the challenges that are faced when studying environmental toxicants in the context of health and disease. For instance, individuals are more likely to be subject to complex mixtures that occur in lower concentrations, resulting in chronic exposure at levels that may not elicit immediate effects. In addition, increases in chemical manufacturing have led to the use of thousands of chemicals lacking adequate toxicity assessments. Thus, a paradigm exists in which the general population is potentially exposed to more compounds through widespread, albeit low, exposures across all stages of development that may alter their disease risk and potentiate adverse health outcomes later in life [100].

#### Viral infection

Viruses, defined as infectious particles comprised of genetic material (DNA or RNA) surrounded by either a protein coat or membrane [101], represent an important component of the environment that is present virtually worldwide. In addition to directly causing diseases such as HIV and shingles, viral infections can increase the risk for a variety of noninfectious diseases, including cancers, allergic diseases, and autoimmune diseases [102–104].

Approximately two million cancer cases annually result from infectious agents, including viruses [105]. Human papillomavirus (HPV), Epstein-Barr virus (EBV), hepatitis B, and hepatitis C (HCV) can cause metastatic transformation of specific cell types originating in a variety of organs. The relationship between infection of high-risk HPV types and anogenital cancers, particularly cervical cancer, is well characterized, with recent studies demonstrating a causal role for HPV in head and neck cancers as well as cancers of the vulva, vagina, penis, and anus [106]. Similarly, HCV infection is associated with hepatocellular carcinoma and subtypes of non-Hodgkin lymphoma, with recent studies suggesting that HCV could also increase the risk of bile duct cancers and diffuse large B-cell lymphoma [107, 108]. EBV infection is associated with several B-cell lymphoproliferative disorders, such as Burkitt lymphoma, Hodgkin disease, systemic non-Hodgkin lymphoma, primary central nervous system lymphoma, and nasopharyngeal carcinoma [109–113]. Some cancers, such as cervical cancer, are related to viral infections acquired during infancy or childhood (for example HPV) that can impact cancer onset later in life. Preventing infections via vaccination can significantly reduce the risk of many of these lethal cancers. A nationwide study in Sweden with over 1.5 million participants showed that quadrivalent HPV vaccine use substantially reduces the risk of invasive cervical cancer [114, 115].

Viral infection can also contribute to the development of multiple allergic diseases [116]. Respiratory viruses have been found to account for 85% of asthma exacerbations in both adults and school-aged children [117]. In particular, respiratory syncytial virus is a risk factor for the development of bronchiolitis and asthma [117–119]. While the exact molecular mechanisms mediating the epidemiological associations of viruses and asthma are not fully understood, they likely involve virus-induced damage of the airways, changes to immune cell activity, modifications to the bacterial microbiome, and additional virulence factors [116]. Causality in association studies is challenged by disease-associated physiological changes in viral defense systems. For example, asthma is associated with rhinovirus infection, and it is challenging to decipher from these statistical associations if patients at risk for asthma have disease-specific physiology that make them more susceptible to rhinoviruses or if rhinovirus infection leads to asthma-specific physiology [120–122].

Viral infections have also been linked to several inflammatory and autoimmune conditions. Some viruses, for example, can induce inflammation that causes tissue damage, as is the case in coxsackievirus B3-induced autoimmune myocarditis [123]. Infection with SARS-CoV-2, the virus that causes coronavirus disease 2019 (COVID-19), has recently been implicated in the development of autoimmune diseases, including Kawasaki disease, pediatric inflammatory multisystemic syndrome, coagulopathy, antiphospholipid syndrome, and Guillain–Barre syndrome [124, 125]. Recent studies also suggest that some severe cases of COVID-19 may be exacerbated by the presence of autoantibodies against type I interferons, meaning that autoimmunity due to autoantibodies made by the adaptive immune system may impair innate antiviral immunity [126]. EBV infection in particular has been associated with a host of autoimmune diseases, including systemic lupus erythematosus (SLE), multiple sclerosis (MS), and rheumatoid arthritis [127–129]. Patients with SLE and MS have a statistically elevated viral load and decreased EBV-driven cell-mediated immunity compared to healthy controls, suggesting that these patients have poorer control over EBV replication [130, 131]. Thus, viral infections have been shown to be powerful drivers of disease, with two recent studies providing highly compelling evidence that EBV infection is causative for MS [132, 133]. Taken together, these examples highlight the need to better understand how viral infections act in concert with disease risk variants to increase risk for diseases with complex etiologies.

#### Climate change

Long-term shifts in temperature and weather patterns due to human activity have both directly and indirectly increased the prevalence of disease [134]. For example, changing temperature and weather patterns are directly accelerating the allergy epidemic by altering concentrations of pollens that exacerbate allergy symptoms [135]. Further, climate change impacts the distribution of vector-borne pathogens, altering the length of transmission seasons and the duration that immunologically naïve populations are exposed to infectious diseases [136]. For example, current models predict that climate change across the world will lead to a climate more suitable for dengue and arbovirus transmission [137]. Indeed, climate change has led to recent outbreaks of dengue, West Nile fever, and chikungunya in Europe [138]. Some countries in sub-Saharan Africa are accustomed to high levels of malaria transmission and thus have developed effective tools to control transmission [137]—such interventions might need to be applied more widely. However, interventions are currently not available for blocking transmission of viruses such as arboviruses and dengue. This leaves populations impacted by climate change-induced virus exposure vulnerable to epidemic-level spread and morbidity.

Climate change can also indirectly increase disease susceptibility by altering socioeconomic factors that leave individuals vulnerable to disease. A recent study showed that warming temperatures and increasing rainfall variability due to climate change adversely affect food security and diet diversity. Such effects are particularly strong in low-income regions, leading to increased malnutrition and impaired childhood development [139, 140]. Thus, the broad impacts of climate change are expected to contribute to disease prevalence both directly and indirectly, highlighting the need to take into consideration how a rapidly changing environment may affect the public health of global communities.

#### Racism, stress, and economic factors

Healthcare disparities are defined as preventable differences in health outcomes that negatively impact groups of people with shared socioeconomic or demographic features. Such differences in disease risk are often driven by environmental exposures [141–145]. Occupational and general environmental exposure to toxicants (e.g., lead in drinking water and paint), rates of nicotine use, and access to high-quality primary care are examples of environmental exposures that impact racial and socioeconomic groups disproportionally [146, 147]. Stress and trauma are well-established environmental risk factors for diseases ranging from heart disease to anxiety that disproportionately impact people who are Black [146, 147]. In a recent study, Resztak et al. developed an approach to derive transcriptional signatures from peripheral blood RNA-seq samples of asthmatic children in the metropolitan Detroit area that were correlated with various psychosocial factors. Among other findings, the authors reported that psychosocial factors altered the expression of 169 genes that have been causally linked to asthma or allergic disease and concluded that the modulation of the immune system may serve as an important mediator between these factors and asthma risk [148]. The deeper implications of this study suggest that molecular-based approaches, when coupled with statistical modeling techniques, could be used to better understand how extrinsic environmental factors may play a disproportionate role in the health and wellbeing of an individual. Taken together, these studies demonstrate that the health of an individual is intricately shaped by their surroundings. Institutional discrimination, which can include socioeconomic status and systemic racism, may dramatically alter the extrinsic factors of an individual’s environment, which may subsequently increase the risk of particular adverse health outcomes. It is therefore imperative for the research community to identify cohorts that are representative of our diverse communities and use current methodologies to identify additional causes of adverse health outcomes. As molecular-based approaches continue to improve, novel techniques may serve as an avenue for identifying previously unknown risk factors, which could then pave the way for developing solutions that would improve public health and close the gap in healthcare disparities.

### Gene × environment interactions

While genetic and environmental factors can independently increase the risk of disease, the interactions between these risk factors (G × E) also have a profound influence on human health. An expanding number of studies have found that disease risk variants impact environmental risk factors, with the implication being that environmental exposures can elicit an altered response in the context of genetic risk variants [149–151]. Identification of G × E interactions and their contributions to disease etiology provides a more comprehensive understanding of the mechanisms driving risk for many human diseases. In this section, we discuss major categories of environmental factors currently implicated in G × E mechanisms.

#### Environmental toxicants

There are many forms of environmental toxicants, and they can influence many diseases. Toxicants represent a special class of G × E interactions because the relationship between the two components is bidirectional—the environment can directly alter the genotype of an individual (i.e., through a somatic mutation), and toxicant metabolism can be affected by inherited genetic variants (i.e., through germline inherited polymorphisms) [152]. Toxicants that cause cancer are called carcinogens. An increase in the amount of carcinogens in the environment has contributed to a global increase in cancer incidence [100]. When carcinogens directly contribute to tumor development, often through a combination of somatic mutations and epigenetic modifications, these changes can directly result in genotype-dependent alterations impacting DNA repair mechanisms or gene regulatory mechanisms [153].

There are numerous ways that environmental toxicants can interact with germline polymorphisms affecting the uptake, metabolism, and transport of toxic compounds. For example, genetic variants associated with arsenic metabolism at the 10q24.32 locus near *AS3MT* are associated with inefficient arsenic metabolism and subsequent toxic arsenic exposure [154]. Similarly, a missense variant in the *FTCD* gene has been proposed to affect the efficiency of arsenic metabolism, potentially by reducing the availability of methyl groups involved in its detoxification [155]. Arsenic contamination in drinking water sources is considered to be a widespread problem and it has been estimated that over 100 million people worldwide are exposed to concentrations exceeding WHO-recommended limits [156]. As a consequence, arsenic is expected to be a significant contributor to disease burden. Paul et al. provide an in-depth review supporting the role of genetic variation in arsenic-induced toxicity, suggesting that the effects of arsenic on the health of an individual have a genotype-dependent component that may account for differences in disease outcome [157]. Like other toxicants, arsenic-related toxicities depend on a multitude of factors including the concentration of the metal, the length of exposure, and the efficiency of its detoxification pathways within the body.

The metabolism of other heavy metals provides additional support for the hypothesis that common polymorphisms contribute to diseases through G × E mechanisms [158]. For example, metallothioneins are metal-binding proteins that regulate metal distribution and help protect cells against heavy metal toxicity. A genetic variant in the core promoter of metallothionein 2A (*MT2A*) affects the expression level of *MT2A*m, which is inversely correlated with the accumulation of cadmium and copper in sinonasal inverted papilloma tissues [159]. Genetic polymorphisms in genes involved in heavy metal metabolism are of significant public health importance because most individuals experience chronic exposure to some level of heavy metals [160].

#### Pollution

The worldwide pollution crisis continues to negatively impact human health. In the example of asthma, both outdoor and indoor air pollution can interact with genetic variants to increase disease risk [161]. A G × E study in mice demonstrated that the magnitude of airway hyperreactivity in response to diesel exhaust particles is dependent upon genotypes at the *Dapp1* locus [162]. In humans, GWAS identified a G × E interaction between diesel exhaust-elicited airway hyperreactivity and a locus on chromosome 3 encoding *DAPP1* [162]. Asthma prevalence is higher among low-income African-American children, who are more likely to reside near highways and industrial areas. The health disparity of asthma can thus be partially attributed to the fact that pollution exposure disproportionately affects low-income populations. Because the currently known genetic and environmental risk factors cannot fully explain the risk of asthma, there is a tremendous need to further delineate additional G × E interactions.

#### Viruses

Viruses interact with their hosts on many levels. When human cells encounter viruses, the pattern recognition and adaptive immune receptors lead to immunological responses aimed at clearing viral infection. Some viruses infect cells without killing them, transitioning to a latent infection. In latency, the virus continues to produce low levels of certain genes, including those encoding transcriptional regulators that interact with the virus and host genomes. The most well-studied GxE viral mechanisms involve viral transcriptional regulatory proteins that interact with the human genome at disease risk variants and alter human gene expression. For example, Epstein-Barr nuclear antigen 2 (EBNA2) regulates human gene expression levels by mimicking activated Notch [163]. Similar to Notch, EBNA2 can influence gene expression by impacting chromatin looping, chromatin accessibility, and human TF binding [164–166]. The genetic locations of these epigenetic effects are highly enriched for autoimmune genetic risk variants. For example, EBNA2 binding events intersect nearly half of known lupus and MS risk loci [52, 164]. Similarly, EBNA2-dependent altered chromatin accessibility and looping events are highly enriched for autoimmune genetic risk variants [164]. Complementary analyses in the same study demonstrate that EBNA2-dependent binding, chromatin accessibility, and chromatin looping at genetic autoimmune disease risk variants are often genotype-dependent [164]. EBNA2 and other EBV proteins are amongst the most highly studied viral transcriptional regulators [167], and it is likely that many other virally encoded transcriptional regulators interact with the human genome at disease risk variants to mediate GxE effects on transcription, cell biology, and disease risk.

#### Genetic modifiers of infectious diseases

In addition to increasing disease risk through interaction with human regulatory elements at disease risk variants, mechanisms initiated by viruses and other infectious agents can also be impacted by rare mutations in key regulators of the immune response. For example, the broad spectrum of disease severity in response to infection with pathogens such as SARS-CoV-2 and influenza is due in part to host genetic variation at loci encoding regulators of antiviral cytokines and innate pattern recognition receptors. Rare mutations in the interferon regulatory factor 7 (*IRF7*) gene, a key regulator of antiviral type I Interferons (IFN-I), have been shown to underlie cases of severe influenza COVID-19 pneumonia. Similarly, whole-exome sequencing on an otherwise healthy child with influenza-induced life-threatening acute respiratory distress syndrome (ARDS) revealed two compound heterozygous mutations in *IRF7*, resulting in very little IFN-I production in response to influenza infection [168]. IRF7 is activated primarily by stimulation of endosomal Toll-Like Receptors (TLRs), resulting in phosphorylation and nuclear localization where IRF7 regulates IFN-I gene expression [169]. Mechanistically, the two mutant loss-of-function alleles result in IRF7 protein that (1) localizes to the nucleus without phosphorylation and (2) does not localize to the nucleus following phosphorylation, respectively. Surprisingly, this patient’s adaptive responses (as measured through B and T cell responses to infection) were normal, suggesting that the life-threatening disease was caused by a blunting of the innate response due to the mutations in the two copies of *IRF7*. In the case of COVID-19, 3.5% of patients with life-threatening COVID-19 pneumonia in one study had genetic defects in TLR-3 and IRF7-dependent signaling pathways of IFN-I [170]. Moreover, deficiencies in the IFN-I pathway are estimated to contribute to nearly 10% of pediatric COVID-19 hospitalizations, despite this age group being classified as low risk for severe disease [171]. These examples illustrate how inherited deficiencies in regulators of the immune response to infection translate to severe outcomes for relatively common diseases.

While some rare loss-of-function mutations can result in life-threatening infection, such effects are likely virus-specific and host-cell intrinsic. A recent study in otherwise healthy humans with inherited IRF7 deficiency showed that while affected individuals were highly susceptible to infections of the respiratory tract, these patients mounted strong immune responses to other pathogens and even retained strong adaptive immune responses to respiratory viruses [172]. Overall, numerous studies have identified specific mutations that can confer risk for severe disease from specific infectious agents, emphasizing the need for future studies that comprehensively identify genetic variants that are impacted by pathogens that can be used to identify patients who are potentially vulnerable.

#### Immunological syndromes and somatic genetic mutations in genes associated with pathogen sensing

The etiology of inflammatory syndromes and diseases that arise in adulthood can be challenging to identify. Somatic genetic mutations that occur after zygote formation have been found to drive some of these complex inflammatory disorders [173]. For example, somatic mutations in *NLRP3*, which encodes an important intracellular sensor of infection, have numerous links to autoinflammatory syndromes [174–178]. Schnitzler’s syndrome is a rare adult-onset autoinflammatory disease that invovles both hematological and rheumatological features, and 90% of patients with Schnitzler’s syndrome who also develop macroglobulinemia carry a somatic mutation in the Toll-like receptor adapter *MYD88* [179]. Patients with Schnitzler’s syndrome who develop a non-malignant expansion of hematopoietic stem cells have somatic mutations in *TET2* and *U2AF1* that are involved in transcriptional and splicing regulation and can impact the production of reactive oxygen species that can trigger the NLRP3-driven inflammasome [180].

With heterogenous symptoms and clinical presentations, recruiting a sufficient number of patients to assess genetic causes of disease is a challenge. Beck et al. addressed this challenge by sequencing the exomes of patients with late-onset inflammatory syndromes that involved peripheral blood abnormalities and were not responsive to treatment [181]. Despite the clinical heterogeneity, somatic deleterious mutations in *UBA1* were identified in a subset of male patients. *UBA1* encodes the E1 enzyme that initiates ubiquitylation, with systemic inflammation resulting from deletion of this gene in zebrafish [181]. Subsequent experiments demonstrated that the myeloid cells (neutrophils and monocytes) and myeloid progenitor stem cells but not the lymphocytes (B and T cells) carried the somatic mutation. In many cases, it is only when the genetic etiology of these inflammatory diseases are appropriately identified that effective treatments are provided. Each of the examples above involve somatic mutations that disrupt sensors and adapters of pathogen detection, and the impact of infection in these patients is yet to be fully elucidated.

With inflammatory disease at the junction between the gut microbiome and human gastrointestinal track, somatic mutations have also been studied in the context of Crohn’s Disease and Ulcerative Colitis. Indeed, somatic mutations in the colonic crypts of patients with these inflammatory bowel diseases are found at a rate 2.4-fold higher than in controls [182]. In particular, an accumulation of somatic mutations in genes known to be important in the pathogenesis of IBD was observed, including those in the IL17 signaling pathway [182].

#### Hormones and sex chromosome composition

An individual’s sex chromosome composition can play a significant role in immune responses and disease severity. However, most pharmaceutical interventions, including most drugs and vaccines, are given without regard to an individual’s sex chromosomes. Because the words male and female can refer to both sex and gender, we focus on chromosomes and hormones in this section of the review. Individuals with two X chromosomes and those with one X chromosome differ in immunological responses to foreign and self antigens. These differences subsequently contribute to variations in susceptibility to infectious diseases, incidence of autoimmune diseases, and responses to vaccines [183]. Indeed, individuals with an XY karyotype are more likely to die from COVID-19 than XX individuals. While such a bias in mortality is consistent with other infections, the specific underlying mechanisms are not fully understood [184]. Moreover, XX individuals typically develop more robust antibody responses and adverse reactions to vaccines [185]. Reasons likely include differences in sex steroid hormones (e.g., estrogen and testosterone) and differences in adaptive immune responses, with XX individuals exhibiting greater antibody responses and elevated humoral and cell-mediated immunity compared to XY individuals. In addition, several immune-related genes encoding proteins such as the IL-2 receptor and multiple Toll-like receptors (TLRs) are encoded on the X chromosome. Epidemiological studies in genetically diverse mice and cohorts of patients with XXX, XXY, or X0 to study the role of sex chromosomes independent of sex hormones have been of great utility for understanding sex chromosome dependent diseases [186–191]. It is critical to consider disease risk and potential G × E mechanisms in the context of sex differences that influence immune responses.

### Possibilities and challenges for studying G × E interactions

The identification and characterization of G × E interactions in humans is crucial to combating human disease [192]. Individuals are born with the genetic variants that they inherit from their parents, and these variants are not easily manipulated. However, many environmental exposures are modifiable or preventable through public policy initiatives, vaccines, and/or lifestyle choices. Likewise, targeting of G × E interactions, for example through genome-editing, holds the promise to enable the development of preventative strategies and therapies. Recent advances in machine learning-based approaches to G × E studies offer one promising solution for learning new G × E mechanisms [148, 193]. However, such methods are still relatively in their infancy, and numerous challenges remain for discovering how the environment works in the context of DNA variation to increase disease risk.

Multiple testing burden is a major challenge in G × E research because of the large sample size required to obtain statistically meaningful associations. A simple solution is to use biologically guided hypotheses to limit the search space, e.g., by limiting analysis to a particular pathway. Another solution is to limit the amount of genetic variation while assessing multiple environmental exposures. For example, a recent study exposed induced pluripotent stem cells derived from six individuals to a variety of treatments to study the environmental effects on allelic gene expression [194]. Because allelic expression was used as a measurement of G × E interactions, a smaller sample size could be used to interrogate environmental exposures. Continued development of additional solutions to the multiple testing problem remains critical.

The environment is difficult to measure and quantify consistently. Many G × E studies address this challenge by developing scores to rank and prioritize environmental exposures. In a recent study, variance quantitative trait locus (vQTL) analysis was performed by associating particular genetic variants associated with phenotypic variability for over 5 million genetic variants in 300,000 individuals. These efforts identified 75 vQTLs highly enriched for G × E effects [195]. This study demonstrates that G × E interactions can be identified without direct measurement of environmental exposures in a large set of samples. The development of additional methodologies will be necessary to quantify specific environmental exposures, identified from large public resources, that increase disease risk.

## Conclusions

The etiology of human disease is complex, with genetic, environmental, and G × E contributors. Studies aimed at genetic or environmental contributors individually can miss important G × E interactions that contribute to disease. A growing body of work has produced compelling evidence linking G × E interactions to a wide range of human diseases. The availability of new datasets with genetic and environmental measurements, in conjunction with the development of novel analytical approaches, will enable the discovery of additional G × E interactions. These discoveries will ultimately lead to impactful interventions that improve human health.
